# Differential Inflammatory-Response Kinetics of Human Keratinocytes upon Cytosolic RNA- and DNA-Fragment Induction

**DOI:** 10.3390/ijms19030774

**Published:** 2018-03-08

**Authors:** Judit Danis, Luca Janovák, Barbara Gubán, Anikó Göblös, Kornélia Szabó, Lajos Kemény, Zsuzsanna Bata-Csörgő, Márta Széll

**Affiliations:** 1 Department of Dermatology and Allergology, University of Szeged, 6720 Szeged, Hungary; janovakluca@gmail.com (L.J.); konczne.guban.barbara.eszter@med.u-szeged.hu (B.G.); goblos.aniko@med.u-szeged.hu (A.G.); kemeny.lajos@med.u-szeged.hu (L.K.); bata.zsuzsa@med.u-szeged.hu (Z.B.-C.); 2 MTA-SZTE Dermatological Research Group, 6720 Szeged, Hungary; szabo.kornelia@med.u-szeged.hu (K.S.); szell.marta@med.u-szeged.hu (M.S.); 3 Department of Medical Genetics, University of Szeged, 6720 Szeged, Hungary

**Keywords:** cytosolic nucleotide fragments, keratinocyte, poly(I:C), poly(dA:dT), signal transduction pathway, interleukin-6, tumor necrosis factor α

## Abstract

Keratinocytes are non-professional immune cells contributing actively to innate immune responses partially by reacting to a wide range of molecular patterns by activating pattern recognition receptors. Cytosolic nucleotide fragments as pathogen- or self-derived trigger factors are activating inflammasomes and inducing anti-viral signal transduction pathways as well as inducing expression of inflammatory cytokines. We aimed to compare the induced inflammatory reactions in three keratinocyte cell types—normal human epidermal keratinocytes, the HaCaT cell line and the HPV-KER cell line—upon exposure to the synthetic RNA and DNA analogues poly(I:C) and poly(dA:dT) to reveal the underlying signaling events. Both agents induced the expression of interleukin-6 and tumor necrosis factor α in all cell types; however, notable kinetic and expression level differences were found. Western blot analysis revealed rapid activation of the nuclear factor κB (NF-κB), mitogen activated protein kinase and signal transducers of activator of transcription (STAT) signal transduction pathways in keratinocytes upon poly(I:C) treatment, while poly(dA:dT) induced slower activation. Inhibition of NF-κB, p38, STAT-1 and STAT-3 signaling resulted in decreased cytokine expression, whereas inhibition of mitogen-activated protein kinase kinase 1/2 (MEK1/2) signaling showed a negative feedback role in both poly(I:C)- and poly(dA:dT)-induced cytokine expression. Based on our in vitro results nucleotide fragments are able to induce inflammatory reactions in keratinocytes, but with different rate and kinetics of cytokine expression, explained by faster activation of signaling routes by poly(I:C) than poly(dA:dT).

## 1. Introduction

The skin provides the primary interface between the body and the environment and forms a physical barrier against invading pathogens. Keratinocytes—the main cell type of the epidermis—form the physical barrier of the stratum corneum and are immunocompetent cells as well, making the epidermis an active member of the immune system.

Keratinocytes express a wide range of pattern recognition receptors and are responsive to various pathogen associated molecular patterns [1,2,3,4], including RNA and DNA fragments, which have been implicated in antiviral defense of keratinocytes [5,6]. Cytosolic RNA and DNA fragments are also known as pathogen- as well as damage-associated molecular patterns (PAMPs and DAMPs), which induce innate immune functions of professional and non-professional immune cells. In non-infectious skin diseases, such as psoriasis, receptors for RNA and/or DNA fragments [7,8,9], moreover their activators: self-derived RNA and DNA fragments and RNA:DNA duplexes are highly abundant in the lesional epidermis [10,11]. During normal cornification, keratinocytes express deoxyribonucleases (DNases) [12]; however, it was recently shown that reduced keratinocyte DNase activity in psoriasis results in suppressed DNA degradation and, as a consequence, parakeratosis [13] and the presence of excess DNA fragments in the cytosol. Similarly, disturbed ribonuclease activities were described in psoriatic skin [14,15], which might result in excess RNA fragments.

These self-derived fragments activate among others the absent in melanoma 2 (AIM2) inflammasome [10,16,17] and inflammatory cytokine expression through their receptors in keratinocytes [7,11,18] initiating inflammatory events. Nucleotide fragment induced reactions have been studied by using synthetic RNA analogue poly(I:C) and DNA analogue poly(dA:dT), which both induce type I interferon (IFN-α/β) and inflammatory cytokine expression in keratinocytes [7,18,19,20]. Poly(I:C) is recognized primarily by toll-like receptor 3 (TLR3) [21], although, TLR3-independent sensing of poly(I:C) has also been observed with involvement of retinoic acid induced gene I (RIG-I) and melanoma differentiation-associated gene 5 [22]. Poly(dA:dT) recognition partially overlaps with poly(I:C) recognition, since RIG-I serves as a receptor after poly(dA:dT) has been transcribed by RNA polymerase III into double-stranded (ds) RNA molecules [23]. Cyclic GMP-AMP synthase (cGAS), a newly described cytosolic DNA receptor implicated in antiviral responses, binds dsDNA sequences independently and activates interferon regulatory factor 3 in cooperation with interferon-γ-inducible protein 16 [6].

The basal expression of most inflammatory cytokines is low and is regulated in response to stimuli at the transcriptional level, mediated by transcription factors of the nuclear factor κB (NF-κB), mitogen activated protein kinases (MAPK) and signal transducers of activator of transcription (STAT) signal transduction pathways [18], which have been reported to participate in nucleotide-induced inflammatory cytokine expression in several cell types [23,24,25,26]. Poly(I:C) was found to induce NF-κB, p38 and STAT-1 signaling in keratinocytes, whereas, in melanocytes, poly(dA:dT) induces NF-κB, p38 and c-Jun N-terminal kinase (JNK) signaling, which differentially regulates cytokine expression [18,24].

Although Cheng and coworkers have reported that sensing of poly(I:C) or poly(dA:dT) and the induced inflammatory reactions after exposure to these molecules partially overlap [22]; no comprehensive data is available for these reactions in keratinocytes. We aimed to compare poly(I:C)- and poly(dA:dT)-induced inflammatory reactions in keratinocytes and the underlying signal transduction events. We found that poly(I:C) and poly(dA:dT) induce similar signal transduction events in keratinocytes; however, the kinetics are faster and the rate of cytokine induction is higher in response to poly(I:C). Moreover, our results suggest a negative feedback role for the activation of extracellular signal-regulated protein kinases 1 and 2 (ERK1/2) signaling in keratinocytes, for both poly(I:C)- and poly(dA:dT)-induced inflammatory signaling.

## 2. Results

### 2.1. Keratinocytes Respond to Poly(I:C) and Poly(dA:dT) with Increased Interleukin-6 (IL-6) and Tumor Necrosis Factor α (TNF-α) Expression

**Figure 1:**
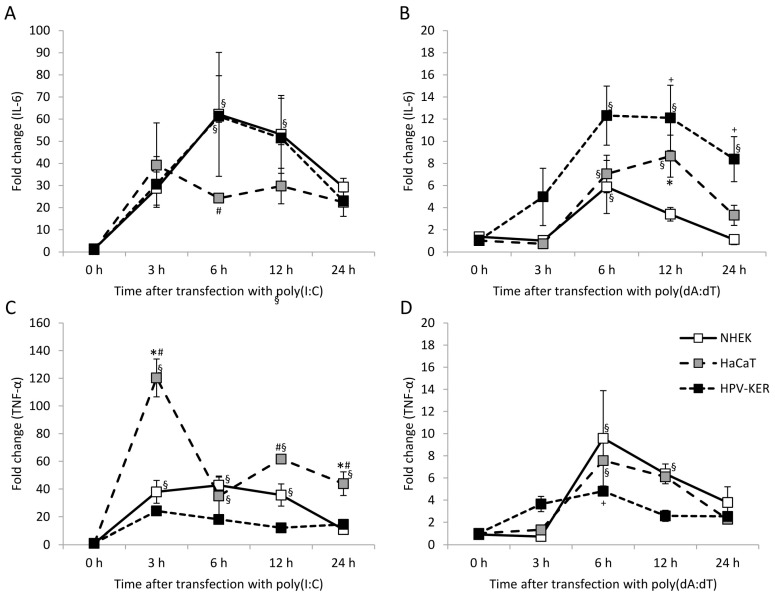
Kinetics of expression of the interleukin-6 (IL-6) and tumor necrosis factor α (TNF-α) cytokines in normal human epidermal keratinocytes (NHEK) and HaCaT and HPV-KER cell lines upon transfection with 0.666 μg/mL poly(I:C) (**A**,**C**) and poly(dA:dT) (**B**,**D**). Relative expression was determined by the ΔΔ*C*_t_ method, normalized to 18S rRNA expression and compared to the expression of the untreated 0 h samples. Data are presented as mean of three independent experiments ± standard error. Significance was determined by two-way repeated measurement analysis of variance (ANOVA), * *p* < 0.05 HaCaT vs. NHEK; ^#^ *p* < HaCaT vs. HPV-KER; ^+^ *p* < 0.05 HPV-KER vs. NHEK; ^§^ *p* < 0.05 vs. 0 h samples within a cell type.

To study cytosolic RNA- and DNA-induced cytokine-expression profiles in keratinocytes, we used three keratinocyte cell types: normal human epidermal keratinocytes (NHEKs), the HaCaT cell line [27] and the HPV-KER cell line [28]. Poly(I:C) strongly induced IL-6 and TNF-α expression in all three cell types, and poly(dA:dT) induced expression in all cell types with slightly different kinetics as well as expression that was an order of magnitude lower than that observed with poly(I:C) (Figure 1). Peak expression was observed 3 to 6 h after poly(I:C) transfection, whereas peak expression after poly(dA:dT) transfection occurred 6 to 12 h after treatment in all cell types studied. Reaction to poly(I:C) in HaCaT cells differed significantly from the other cell types (Figure 1A,C). In contrast, poly(dA:dT)-induced reaction differed in all three cell types (Figure 1B,D). Previously we found that the HPV-KER cell line and NHEK cells exhibited similar cytokine mRNA expression [28], which agreed with our finding on the expression kinetics upon poly(I:C) induction.

To study the induced signaling pathways in keratinocytes, we used only the HPV-KER cell line. HPV-KER cells previously showed similar reactions to NHEKs [28] and HaCaT cells exhibited a slightly different cytokine-expression profile, moreover HaCaT cells are known to exhibit constant activation of inflammatory signaling [29], while high intra-individual differences were observed in the inflammatory inductions of NHEKs.

### 2.2. Poly(I:C) and Poly(dA:dT) Treatment Induces Nuclear Factor κB (NF-κB), Mitogen Activated Protein Kinase (MAPK) and Signal Transducers of Activator of Transcription (STAT) Activation in Keratinocytes

**Figure 2:**
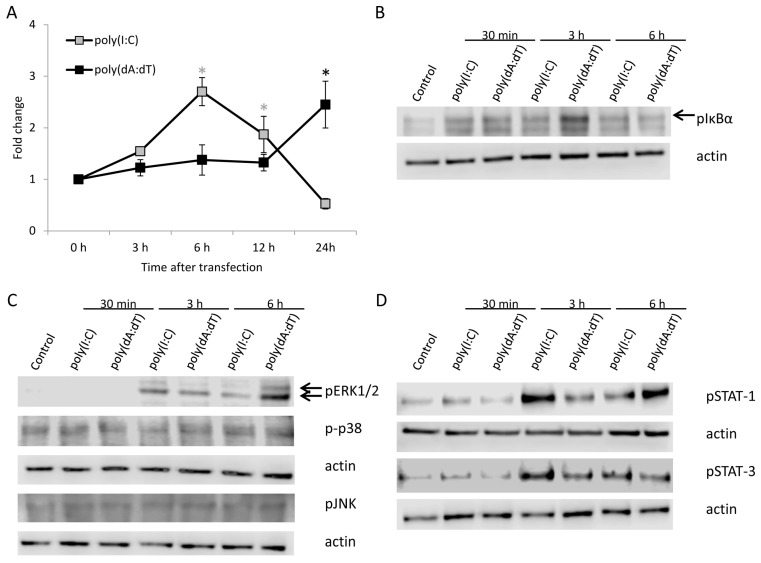
Activation of Nuclear Factor κB (NF-κB), Mitogen Activated Protein Kinase (MAPK) and Signal Transducers of Activator of Transcription (STAT) signal transduction pathways in HPV-KER cells upon poly(I:C) or poly(dA:dT) transfection assessed by NF-κB-luciferase reporter assay (**A**) and western blot analysis (**B**–**D**). (**A**) NF-κB luciferase reporter assay exhibited faster activation of NF-κB transcription factor upon poly(I:C) treatment than poly(dA:dT) treatment. Raw luminescence intensity values were normalized to the intrinsic control renilla activity, and compared to the 0 h untreated samples. Data are presented as mean of three independent experiments ± standard error; statistical significance was assessed by two-way repeated measurement ANOVA * *p* < 0.05, grey: poly(I:C) treatment compared to untreated 0 h samples, black: poly(dA:dT) treatment compared to untreated 0 h samples; (**B**) Increase in phosphorylated NF-κB inhibitor α (IκBα) after poly(I:C) or poly(dA:dT) treatment, peaking later after poly(dA:dT) treatment than after poly(I:C) treatment, arrow indicate the lane for phosphorylated IκBα; (**C**) Phosphorylation of ERK1/2 increases after poly(I:C) or poly(dA:dT) treatment, peaking later after poly(dA:dT) treatment than after poly(I:C) treatment, arrows indicate from top to bottom the lanes for phosphorylated ERK1 and ERK2. Phosphorylation of p38 and JNK was not observed upon poly(I:C) or poly(dA:dT) treatment; (**D**) Phosphorylation of both STAT-1 and STAT-3 occurs faster in poly(I:C) treated samples than in poly(dA:dT) treated samples. Western blot results are representative for at least three independent experiments. Actin was used as loading control.

NF-κB activation in HPV-KER keratinocytes was assessed by an NF-κB–luciferase reporter assay (Figure 2A). The kinetic differences of NF-κB activation between poly(I:C) and poly(dA:dT) transfected cells resembled the corresponding cytokine expression differences: peak-activation occurred at 6 h after poly(I:C) treatment, whereas the peak activation with poly(dA:dT) occurred 24 h after treatment. The delayed NF-κB signaling response to poly(dA:dT) was confirmed with detection of phosphorylated NF-κB inhibitor α (IκBα) by western blot analysis (Figure 2B and Figure S1A).

Western blot analysis of MAP kinase (Figure 2C) and STAT (Figure 2D) pathways showed that both poly(I:C) and poly(dA:dT) induced the phosphorylation of ERK1/2 and STAT-1 as well as STAT-3 signaling. Densitometric analysis showed a faster phosphorylation of STAT-1 and STAT-3 in poly(I:C) treated samples compared to poly(dA:dT) treatment (Figure S1). In addition, phosphorylation of p38 MAPK and JNK pathways were not affected at the studied time points, which was also confirmed by densitometric analysis (Figure S1C,D).

### 2.3. Cytokine Expression of Keratinocytes upon Poly(I:C) and Poly(dA:dT) Treatment Relies on NF-κB, p38 and STAT Signaling

To address the role of the activated signaling routes in poly(I:C)- and poly(dA:dT)-induced cytokine expression, keratinocytes were pre-incubated with the specific inhibitors of NF-κB (Bay 11-7085), dual specificity mitogen-activated protein kinase kinase1 and 2 (MEK1/2) (PD95089), p38 (SB203580), JNK (SP600125), STAT-1 (fludarabine) and STAT-3 (Stattic) for an hour before transfection with poly(I:C) or poly(dA:dT).

**Figure 3:**
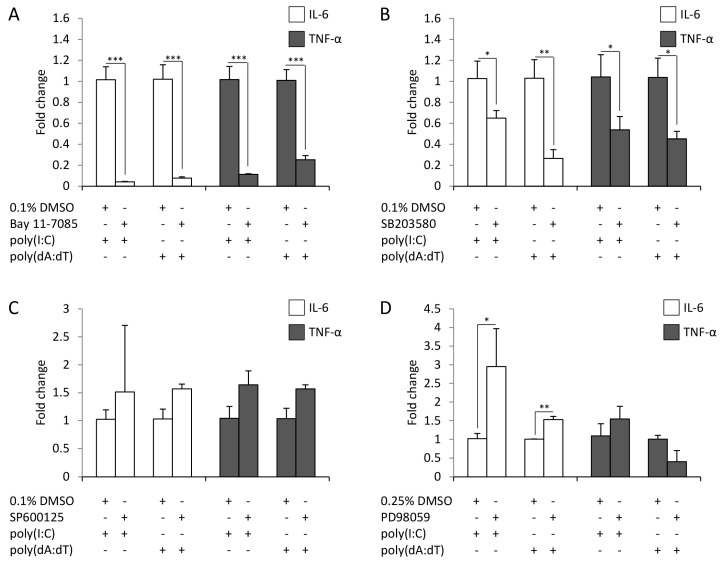

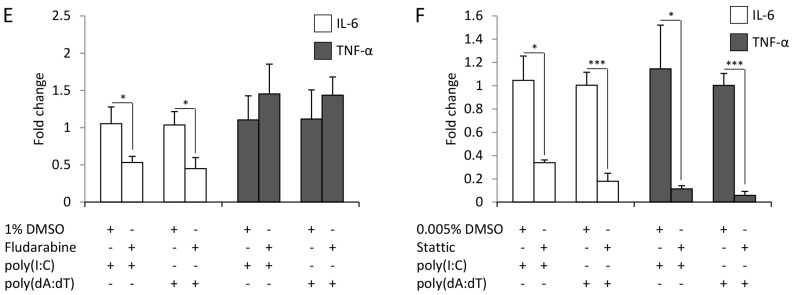
Inhibition of different signaling routes has divergent effects on the expression of the IL-6 (white bars) and TNF-α (grey bars) cytokines in keratinocytes. The effect of inhibition by NF-κB (**A**), p38 (**B**), c-Jun N-terminal kinase (JNK) (**C**), mitogen-activated protein kinase kinase 1 and2 (MEK1/2) (**D**), STAT-1 (**E**) and STAT-3 (**F**) on poly(I:C) (6 h after transfection) and poly(dA:dT) (12 h after transfection) induction of IL-6 (white bars) and TNF-α (grey bars) expression in HPV-KER cells. Fold change of mRNA expression values were determined by the ΔΔ*C*_t_ method, normalized to 18S rRNA expression. As all inhibitors were dissolved in dimethyl sulfoxide (DMSO), the relative mRNA expression levels were compared to the expression levels in samples treated with DMSO + poly(I:C) or DMSO + poly(dA:dT), respectively. Poly(I:C) and poly(dA:dT) induction was in every case significant compared to the untreated control samples; no significant difference was observed between the cytokine-expression level of the samples treated with poly(I:C), poly(dA:dT), DMSO + poly(I:C) or DMSO + poly(dA:dT). Data are represented as the means of three independent experiments ± standard error; * *p* < 0.05; ** *p* < 0.01; *** *p* < 0.001 determined by Student’s *t*-test.

Time points of sample collection were determined with respect on the peak expression of cytokines (Figure 1). Inhibition of NF-κB nearly completely abolished both the poly(I:C)- and poly(dA:dT)-induced expression of IL-6 and TNF-α (Figure 3A).

Although activation could not be confirmed by our western blot results (Figure 2C), inhibition of p38 signaling resulted in significantly decreased IL-6 and TNF-α expression (Figure 3B). In contrast, the inhibition of JNK signaling did not affect cytokine expression (Figure 3C). The inhibition of MEK-1 signaling significantly increased the poly(I:C)- and poly(dA:dT)-induced production of IL-6 (Figure 3D), suggesting a possible negative regulatory role of this pathway.

Specific inhibition of STAT-3 signaling significantly decreased both poly(I:C)- and poly(dA:dT)-induced cytokine expression (Figure 3F), whereas the inhibition of STAT-1 affected only IL-6 expression (Figure 3E). Thus, whereas both poly(I:C)- and poly(dA:dT)-induced IL-6 expression was affected by most of the studied signaling routes, TNF-α expression was only affected by NF-κB, p38 and STAT-3.

## 3. Discussion

RNA and DNA fragments are known as important PAMPs or DAMPs that induce innate immune processes of professional immune cell types, such as macrophages and dendritic cells [23,30,31,32], as well as non-professional immune cells, such as keratinocytes [18]. Accumulation of nucleotide fragments in keratinocytes is involved in the pathogenesis of psoriasis leading to parakeratosis [13], as well as in the promotion of inflammation by activating dendritic cells [33] and in the activation of inflammasomes in keratinocytes [10,17]. However, the induced inflammatory signaling pathways and how they contribute to cytokine expression in keratinocytes were not previously studied.

In this study, we characterized innate immune responses of human keratinocytes to the cytosolic exposure of the dsRNA analogue poly(I:C) and the dsDNA analogue poly(dA:dT). We compared the IL-6 and TNF-α expression of NHEK, HaCaT [27] and HPV-KER [28] cells after poly(I:C) and poly(dA:dT) exposure. Previously we found that the inflammatory responses of the widely used HaCaT cell line upon exposure to *Propionibacterium acnes* differ from that of NHEKs, while the HPV-KER cell line (established and characterized in our laboratory) shows similar inflammatory [28,34] and ultraviolet-B irradiation-induced responses to NHEKs [35]. In line with our previous findings [28], we found that the cytokine expression patterns in the HaCaT cells differ significantly from the responses of HPV-KER and NHEK cells. Moreover, HaCaT cells are thought to be less suitable to study inflammatory signaling pathways due to their constant NF-κB activation [29].

**Figure 4:**
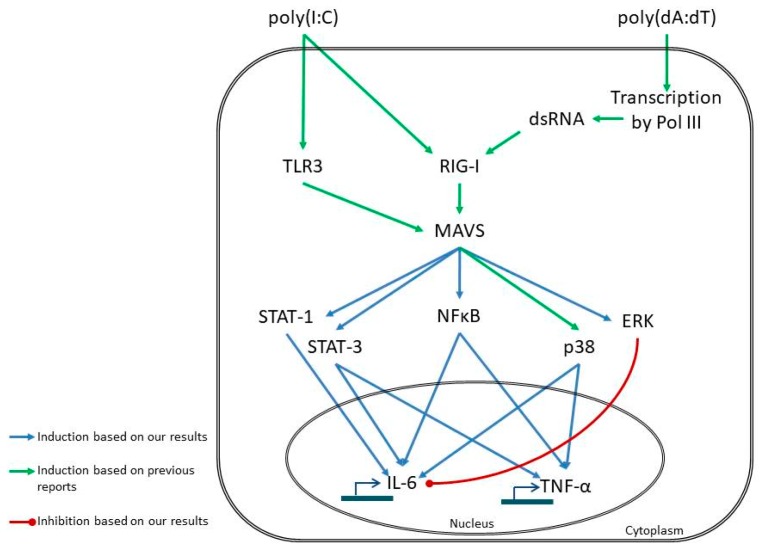
Proposed model of poly(I:C)- and poly(dA:dT)-induced signaling events leading to IL-6 and TNF-α expression in keratinocytes, based on our results and results of previous reports [21,22,23,44]. Our results indicate that poly(I:C) and poly(dA:dT) induce the same inflammatory pathways; however, the mode of sensing differs, leading to the observed differences in kinetic. The receptors for poly(I:C) sensing are toll-like receptor 3 (TLR3) [21] and retinoic acid induced gene I (RIG-I) [22]. In contrast, poly(dA:dT) is transcribed to double-stranded (ds) RNA by RNA polymerase III (Pol III) and is subsequently sensed by RIG-I [23]. The transcription step might be responsible for the delayed response to poly(dA:dT). The receptors activate the NF-κB, MAPK and STAT signaling routes through the adaptor molecule mitochondrial antiviral signaling protein (MAVS), and, thus, regulate the transcription of the cytokines.

NF-κB, MAPKs and STAT signaling have been reported to participate in nucleotide-fragment-induced inflammatory cytokine expression in several cell types [22,23,24,25,26]; however, limited information is available for these signaling events in keratinocytes upon nucleotide fragment induction [18,36]. According to our results, poly(I:C) induces activation of the studied signaling pathways in a shorter time than poly(dA:dT), and a corresponding shift in cytokine expression peaks was observed. The difference in peak timing is likely due to direct activation of TLR3 signaling by poly(I:C) [37]; while it has been shown that poly(dA:dT) must first be transcribed to RNA before activating NF-κB through RIG-I dependent sensing [23] (Figure 4).

In addition to poly(I:C)- and poly(dA:dT)-induced NF-κB activation, phosphorylation of ERK1/2, STAT-1 and STAT-3 was also observed; but phosphorylation of other studied MAPKs (p38 and JNK) was not affected. In contrast, a previous study using keratinocytes reported poly(I:C) induction of p38 signaling but no induction of ERK1/2 signaling [18]. In melanocytes, another epidermal cell type, poly(dA:dT)-induced phosphorylation of p38 and JNK signaling was observed without ERK1/2 activation [24]. These differences might be due to differences in time points used: our study examined p38 phosphorylation 30 min after poly(I:C) and poly(dA:dT) transfection in HPV-KER cells, whereas the previous study examined p38 phosphorylation in NHEKs 15 min after treatment, observing a reduction after 30 min [18]. These results suggest that poly(I:C)- and poly(dA:dT)-induced p38 phosphorylation might be a rapid event in keratinocytes. Although we could not confirm p38 phosphorylation, inhibition of p38 signaling during transfection with poly(I:C) or poly(dA:dT) resulted in decreased IL-6 and TNF-α expression, which is in agreement with a previous report on poly(I:C)-induced TNF-α expression in keratinocytes [18].

In monocytes and melanocytes, inhibition of ERK1/2 and JNK signaling pathways abolished nucleotide-induced IL-6 and TNF-α expression [24,38]. In mouse models, the disruption of ERK1/2 signaling by the inhibition of MEK1/2 functions have been shown to have anti-inflammatory effects [39,40]. In contrast, in our experiments the disruption of ERK1/2 signaling through inhibition of MEK1/2 kinases increased the expression of the inflammatory mediator IL-6. These results agree with previous in vivo findings that therapeutic inhibition of MEK1/2 in patients is often accompanied by an inflammatory skin rash [41]. These results suggest that ERK1/2 signaling—in contrast to other cell types—has a negative regulatory function in inflammatory reactions in keratinocytes. Previous reports have already demonstrated similar results: ERK1/2 signaling was shown to negatively regulate NF-κB activation [42], and inhibition of MEK1/2 led to increased NF-κB, STAT-1 and interferon-regulatory factor signaling in human keratinocytes [43], altough, we did not observe an increase in NF-κB activation upon inhibition of ERK1/2 signaling.

STAT signaling is known to be induced by inflammatory cytokines [45] and by poly(I:C) treatment [18,36]. We found that, in addition to poly(I:C), poly(dA:dT) also induced STAT-1 and STAT-3 signaling in keratinocytes, and that the induction exhibited a delay in activation after poly(dA:dT) transfection similar to those observed with other pathways. Poly(I:C)-induced STAT-1 activation has been shown to regulate TLR3 and TNF-α expression [18]. In our experiments, STAT-1 was found to regulate poly(I:C)- and poly(dA:dT)-induced IL-6 expression, whereas TNF-α was not affected. Inhibition of STAT-3 abolished both poly(I:C)- and poly(dA:dT)-induced IL-6 and TNF-α expression, showing the different regulatory functions of each STAT transcription factors.

In this study, we show the similarities as well as the differences in inflammatory signaling events of keratinocytes induced by dsRNA and dsDNA. Our data revealed that transfection with the synthetic dsRNA and dsDNA analogues poly(I:C) and poly(dA:dT) induced activation of NF-κB and STAT signaling, both of which were also shown to be functional in inducing cytokine expression. Moreover, we showed the negative regulatory role of ERK1/2 signaling in nucleotide-induced cytokine expression. Although dsRNA and dsDNA are recognized by different sets of receptors, they induce the same inflammatory signaling pathways in keratinocytes, albeit with different kinetics and magnitude of activation.

Studies of the last decade have highlighted disturbances in the signal transduction events in psoriasis that have led to the development of targeted therapeutics against specific signaling components. However there is still a lack of knowledge on every aspect of these mechanisms. Our results deepen the existing knowledge and contribute to the understanding of these signaling events induced in keratinocytes.

## 4. Materials and Methods

### 4.1. Cell Culture

NHEK cells, the HaCaT cell line [27] and the HPV-KER cell line established in our laboratory [28] were used for the experiments. After obtaining written informed consent from investigated individuals, skin speciments from the Plastic Surgery Unit of our Department were used to separate NHEKs, as described previously. Investigations were carried out in accordance with the rules of the Helsinki Declaration, and prior study, the study design was approved by the Human Investigation Review Board of the University of Szeged (PSO-EDAFN-002, 23 February2015, Szeged, Hungary). The epidermis was separated from the dermis with overnight incubation in Dispase (Roche Diagnostics, Manheim, Germany), and keratinocytes were obtained after maceration in 0.25% trypsin. All cell types were grown in 75 cm 2 cell culture flasks. NHEKs and HPV-KER cells were maintained in keratinocyte serum-free medium containing epidermal growth factor and bovine pituitary factor (Gibco Keratinocyte SFM Kit; Life Technologies, Copenhagen, Denmark) and supplemented with 1% antibiotic/antimycotic solution (PAA Laboratories GmBH, Pasching, Austria) and 1% l-glutamine (PAA Laboratories). HaCaT cells were grown in DMEM with 4.5 g/L glucose supplemented with 1% antibiotic/antimycotic solution, 1% l-glutamine and 10% fetal bovine serum at 37 °C in a humidified atmosphere with 5% CO_2_. The medium was changed every 2 days.

### 4.2. Stimulation of the Cells

HPV-KER cells, HaCaT cells or third passage NHEKs were seeded into 6-well plates. After 24 h, cells were transfected with 0.666 μg/mL polydeoxyadenylic acid-polydeoxythymidylic acid double-stranded homopolymer (poly(dA:dT)) (InvivoGene, San Diego, CA, USA) or with 0.666 μg/mL polyinosinic-polycytidylic acid (poly(I:C)) (Sigma Aldrich, Saint Louis, MO, USA) using the X-tremeGene 9 transfection reagent (Roche Diagnostics). Cells were harvested at indicated time points.

For inhibition studies, cells were incubated 1 h prior to poly(dA:dT)/poly(I:C) transfection with inhibitors for NF-κB (Bay 11-7085, 10 μM; MedChem Express, Monmouth Junction, NJ, USA), STAT-1 (Fludarabine, 10 μM; Sigma Aldrich), STAT-3 (Stattic, 5 μM; Sigma Aldrich), MEK1 (PD98059, 20 μM; Sigma Aldrich), JNK (SP600125, 10 μM; Tocris Bioscience, Bristol, UK) and p38 (SB203580, 10 μM; Tocris Bioscience).

### 4.3. RNA Isolation and RT-PCR

Cells were harvested in TRIzol^®^ Reagent (Invitrogen Corp., Carlsbad, CA, USA) and total RNA was isolated following the manufacturer’s instructions. Potential genomic DNA contamination was removed by using the Turbo DNA-free Kit (Ambion, Life Technologies) according to the manufacturer’s instructions. 1 µg total RNA was reverse transcribed into cDNA by the iScript cDNA Synthesis Kit (Bio-Rad Laboratories, Hercules, CA, USA). Real-time RT-PCR experiments were carried out with the Universal Probe Library system TaqMan probes (Roche Diagnostics) and qPCRBIO Probe Mix Lo-ROX (PCR Biosystem Ltd., London, UK) on a C1000 Touch Thermal Cycler (Bio-Rad Laboratories), using primers reported previously [20]. The expression of each gene was normalized to the expression of the 18S rRNA gene. Relative mRNA levels were calculated by the ΔΔ*C*_t_ method.

### 4.4. Detection of NF-κB Induction

Luciferase assays were performed to determine the NF-κB activity in response to poly(dA:dT) treatment. The HPV-KER cell line was transfected with the NF-κB reporter construct vector pNF-κB-luc *Cis*-Reporter Plasmid (Stratagene, La Jolla, CA, USA) and the pGL4.75 [hRluc/CMV] plasmid (Promega, Madison, WI, USA) with the use of the X-tremeGene9 transfection reagent. The treated cells were washed twice with PBS, lysed with passive lysis buffer (Biotium, Hayward, CA, USA) and the luciferase activities in the lysates were measured using the Firefly & Renilla Dual Luciferase Assay Kit (Biotium) and Thermo Luminoskan Ascent (Thermo Scientific, Rockford, IL, USA), according to the manufacturer’s instructions. All samples were measured three times and the luciferase activity derived from the NF-κB-luc plasmid was normalized to the activity of the Renilla luciferase activity from pGL4.75 [hRluc/CMV] plasmid.

### 4.5. Western Blot Analysis

Cells were harvested at indicated time points after poly(dA:dT) or poly(I:C) transfection and lysed in lysis buffer supplemented with 0.5% SDS and 1% Halt™ Protease and Phosphatase Inhibitor Cocktail (Thermo Scientific). Equal amounts of protein in ProTrack Loading Buffer (Lonza, Basel, Switzerland) were separated on a 7.5% TGX Fast Cast Gel and transferred to nitrocellulose membrane (0.45 μm; Bio-Rad Laboratories). After blocking the membrane in 5% non-fat milk in Tris-Buffered Saline containing 0.2% Tween-20, primary antibodies were incubated overnight at 4 °C with constant agitation. HRP-conjugated secondary antibodies were incubated for 60 min at room temperature. Signal was visualized with SuperSignal™ West Pico Chemiluminescent Substrate (Thermo Scientific) on a C-Digit Blot Scanner (LI-COR Corp., Lincoln, NE, USA). Primary antibodies used were phospho-IκBα (Santa Cruz Biotechnology, Dallas, TX, USA), phospho-ERK1/2 (BioLegend, San Diego, CA, USA), phospho-STAT-1 (Ser727; Cell Signaling Technology, Danvers, MA, USA), phospho-STAT-3 (Ser727; Cell Signaling Technology), phospho-JNK (T183/Y185; Bio-Techne, Abingdon, UK) and phospho-p38 alpha (T180/Y182; Bio-Techne).

### 4.6. Statistical Analysis

Two-way repeated measurement analysis of variance (ANOVA) was used to compare more than two groups, and one-tailed, paired Student’s *t*-test was used to compare two groups, as indicated in the figure legend. Based on at least three independent biological repeats, data are presented as mean ± standard error. Statistical analysis was carried out using Sigma Plot Ver. 13.0, (Systat Software Inc. Erkrath, Germany) the significance level was set at *p* ≤ 0.05.

## Supplementary Materials

Supplementary materials can be found at http://www.mdpi.com/1422-0067/19/3/774/s1.

## Abbreviations

IL-6	interleukin-6
TNF-α	tumor necrosis factor α
PAMP	pathogen associated molecular pattern
DAMP	damage associated molecular pattern
DNase	deoxyribonuclease
AIM2	absent in melanoma 2
IFN	interferon
TLR	toll like receptor
RIG-I	retinoic acid induced gene I
cGAS	cyclic GMP-AMP synthase
ds	double-stranded
poly(dA:dT)	polydeoxyadenylic acid-polydeoxythymidylic acid double-stranded homopolymer
poly(I:C)	Polyinosinic-polycytidylic acid
MAPK	mitogen activated protein kinase
JNK	c-Jun N-terminal kinase
ERK1/2	extracellular signal-regulated protein kinase 1 and 2
MEK1/2	dual specificity mitogen-activated protein kinase kinase 1 and 2
NF-κB	nuclear factor κB
IκBα	NF-κB inhibitor α
STAT	signal transducer and activator of transcription
NHEK	normal human epidermal keratinocyte
