# Supplementary material for: Differential Inflammatory-Response Kinetics of Human Keratinocytes upon Cytosolic RNA- and DNA-Fragment Induction
Source: Int J Mol Sci. 2018 Mar 8;19(3):774. doi: 10.3390/ijms19030774 (PMC5877635; doi:10.3390/ijms19030774)
Supplement: Supplementary file 1 [file ijms-19-00774-s001.pdf]

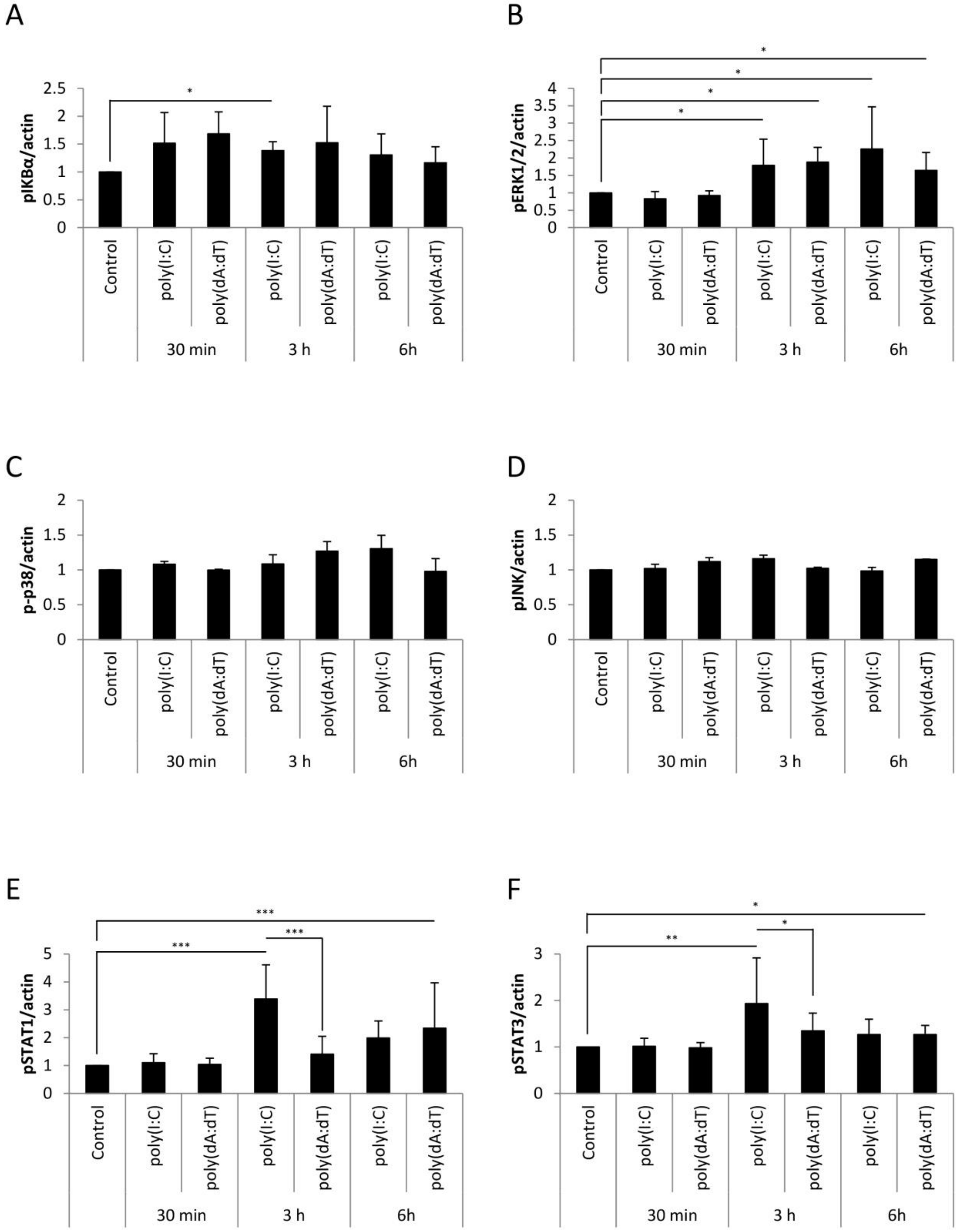

Supplementary Figure 1. Densitometric analysis of Western blots on the phosphorylated forms IκBα (A), ERK1/2 (B), p38 (C), JNK (D), STAT-1 (E), STAT3 (F) normalized to the level of loading control actin. Signal was visualized with SuperSignal™ West Pico Chemiluminescent Substrate (Thermo Scientific) on a C-Digit Blot Scanner (LI-COR Corp. Lincoln, NE, USA), densitometric analysis of the bands was carried out using Image Pro Plus software (Media Cybernetics Inc. Rockville, MD, USA). Intensity of bands from the protein of interest were normalized to the intensity of actin bands of the respective blots. Statistical analysis was carried out by two-way repeated measurement ANOVA (Sigma Plot Ver. 13.0 Systat Software Inc. Erkrath, Germany). Data are presented as means ± SD of the densitometric analysis of n=5 (A,B,E,F) or n=3 (C,D) blots. *$p<0.05$; **$p<0.01$; ***$p<0.001$
